# Aerobic Anoxygenic Phototrophic Bacteria Promote the Development of Biological Soil Crusts

**DOI:** 10.3389/fmicb.2018.02715

**Published:** 2018-11-13

**Authors:** Kai Tang, Lijuan Jia, Bo Yuan, Shanshan Yang, Heng Li, Jianyu Meng, Yonghui Zeng, Fuying Feng

**Affiliations:** ^1^Institute for Applied and Environmental Microbiology, College of Life Science, Inner Mongolia Agricultural University, Huhhot, China; ^2^College of Life Science, Inner Mongolia Normal University, Huhhot, China; ^3^Aarhus Institute of Advanced Studies, Aarhus, Denmark; ^4^Department of Environmental Science, Aarhus University, Roskilde, Denmark

**Keywords:** AAnPB, biological soil crusts, *puf*M, co-occurrence networks, *Methyloversatilis*, *Sphingomonas*

## Abstract

Chlorophyll-containing oxygenic photoautotrophs have been well known to play a fundamental role in the development of biological soil crusts (BSCs) by harvesting solar radiations and providing fixed carbon to the BSCs ecosystems. Although the same functions can be theoretically fulfilled by the widespread bacteriochlorophyll-harboring aerobic anoxygenic phototrophic bacteria (AAnPB), whether AAnPB play a role in the formation of BSCs and how important they are to this process remain largely unknown. To address these questions, we set up a microcosm system with surface sands of the Hopq desert in northern China and observed the significant effects of near-infrared illumination on the development of BSCs. Compared to near-infrared or red light alone, the combined use of near-infrared and red lights for illumination greatly increased the thickness of BSCs, their organic matter contents and the microalgae abundance by 24.0, 103.7, and 1447.6%, respectively. These changes were attributed to the increasing abundance of AAnPB that can absorb near-infrared radiations. Our data suggest that AAnPB is a long-overlooked driver in promoting the development of BSCs in drylands.

## Introduction

Global drylands cover about 41% of the Earth’s terrestrial surface with arid, semiarid and dry subhumid areas (Feng and Fu, 2013). These regions are expanding on an appreciable scale, due to climate changes, deterioration in water supplies and land degradation (Hallenbeck, 2017). In arid and semi-arid lands, biological soil crusts (BSCs) are widely distributed as one of the major features of the Earth’s terrestrial surface. BSCs can help in maintaining soil fertility and reducing erosion, and thus play a vital role in global carbon (Elbert et al., 2012; Porada et al., 2013, 2014) and nitrogen cycling (Belnap, 2003). BSCs consist of cyanobacteria, eukaryotic microalgae, microfungi, lichens, bryophytes and heterotrophic bacteria, among which phototrophs play a crucial role in the development of BSCs (Belnap et al., 2016). Cyanobacteria are one of the key players in the development of BSCs, as they can enhance soil nutrition content through fixation of atmospheric carbon and nitrogen and by secretion of polysaccharide that can condense sand particles (Belnap et al., 2016; Hallenbeck, 2017). In contrast, very little is known about the presence and potential function of aerobic anoxygenic phototrophic bacteria (AAnPB) in BSCs, another important and widespread group of phototrophs in nature using bacteriochlorophyll *a* (BChl *a*) as the major photosynthetic pigments (Koblížek, 2015; Yurkov and Hughes, 2017).

Aerobic anoxygenic phototrophic bacteria perform anoxygenic photosynthesis exclusively under aerobic conditions. They have been reported to exist in BSCs and could accelerate organic carbon cycling in nutrient-poor arid soils (Csotonyi et al., 2010), suggesting that light and oxygen rich BSCs might be a suitable niche for AAnPB, although BChl *a* were often failed to be detected in BSCs (Nagy et al., 2005). Whether AAnPB play a role in the development of BSCs and how important they are to this process remain elusive.

While chlorophylls in cyanobacteria and other oxygenic photoautotrophs absorb light primarily at 514–700 nm, BChl *a* in AAnPB can absorb near-infrared (NIR) light at 760–1130 nm with minimum absorption at the red part of visible light (Stomp et al., 2007). This difference in absorption spectra was due to various modifications to chlorophylide *a*, the hub metabolite that is further used for biosynthesis of all types of chlorophylls and bacteriochlorophylls (Chew and Bryant, 2007) in various phototrophic microorganisms (Zeng et al., 2014; Thiel et al., 2018). In the atmosphere, absorption of incident solar NIR mostly occurs when NIR interacts with water vapors, clouds and greenhouse gasses. Considering the extreme paucity of moisture and clouds in the air above drylands, it was reasonable to speculate that BSCs in drylands receive relatively abundant solar NIR radiations. At a Gobi desert field site in northern China, the NIR radiations accounted for an average of 54.6% in total solar radiations (UV-Visible-NIR) during a 1-year measurement (Zheng et al., 2015), significantly higher than the average level of ca. 40% at the whole Earth’s surface based on a model study (Hatzianastassiou et al., 2005).

We hypothesized that these solar NIR radiations can be absorbed by AAnPB inhabiting BSCs and aid their growth. Certain species of AAnPB have demonstrated the capabilities of fixing atmospheric nitrogen (Yurkov and Csotonyi, 2009), carbon dioxide (Bill et al., 2017), and producing polysaccharide (Tahon et al., 2016). These functional traits similar to those of cyanobacteria might contribute to the soil conservation by promoting the development of BSCs, especially in the dry lands stressed with low nutrient level. To test this hypothesis, we set up microcosm systems in the laboratory with surface sand soils of bare sand dunes collected from a desert in northern China. Three illumination treatments were employed, i.e., red light, NIR and red plus NIR. Over a course of 120-day incubation, we observed significant changes in soil surface appearance, their physiochemical properties and the composition of microbial communities. These changes were tightly correlated with the increase in the abundance of AAnPB. Thus, we demonstrated that AAnPB could promote the development of BSCs and play a long overlooked role in the assembly of functional microbial communities in BSCs. This finding could have an impact on the practice of soil fertilization and bioremediation in drylands.

## Materials and Methods

### Microcosm Experiments Design and Soil Sample Collection

**FIGURE 1:**
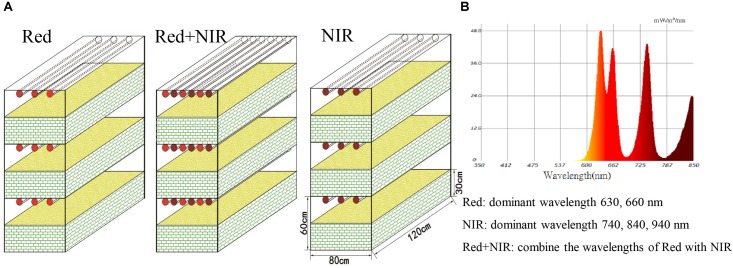
Microcosmic systems **(A)** and light composition **(B)**. The experiment was set up in three treatments illuminated by Red (range from 560 to 700 nm, peak light wavelengths at 630 and 660 nm), NIR (range from 700 to 1000 nm, peak light wavelengths at 740, 840, and 940 nm) and Red + NIR (range from 560 to 1000 nm, peak light wavelengths at 630, 660, 740, 840, and 940 nm), and in three replicates (three layers in each treatment), respectively. The luminous intensity of Red and Red + NIR was approximately 3,000 lux at each layer (single NIR was approximately 70 lux at each layer). Each box in this microcosmic experiment was 0.80 m wide, 1.20 m long, and 0.30 m tall, and was filled with sand soils. The distance from sand top to the led lamp was 0.30 m.

Bacteriochlorophylls in AAnPB absorb NIR but not red light while chlorophylls in cyanobacteria and other algae absorb red light but not NIR. Therefore, we used these two types of light sources to test the eco-physiological functions of AAnPB in the development of BSCs. Dry and soft surface sand soils (5 – 10 cm) of bare sand dunes were collected in the eastern margin of the Hopq desert (40°4′ N, 110° 47′E) in northern China on 5 March, 2015 and transported to our laboratory in Huhhot within the same day. In the laboratory, sands were mixed and loaded into a bottom-side-breathable plastic box (0.8 × 1.2 m^2^) and placed in a dark room. We set up three illumination treatments, i.e., Red (range from 560 to 700 nm, peak light wavelengths at 630 and 660 nm), NIR (range from 700 to 1000 nm, peak light wavelengths at 740, 840, and 940 nm), and Red + NIR (range from 560 to 1000 nm, peak light wavelengths at 630, 660, 740, 840, and 940 nm) using custom-made LED lamps (Figure 1). Each setup was done in triplicate. The luminous intensity of Red and Red + NIR was ca. 3000 lux at each layer, while NIR alone was approximately 70 lux at each layer. An additional test on 140 lux NIR was done on a Red + NIR setting to observe the dose effect of NIR on BSCs development. The soils were incubated with a constant relative humidity of 60–70% under 12 h/12 h-light-dark cycles at room temperature (22.5 ± 2°C). BSCs were well known to develop extremely slowly in natural environments. To speed up this process and to avoid unexpected contaminations that may be introduced from indoor environments during a very long incubation, each box was sprayed with 25 L of sterilized distilled-water by an eclectic pump in the beginning and then, was watered with 15 L every 30 days. No watering was performed at least 14 days before sampling. The water content of sampled soils was about 7.5%.

A few grams of BSCs of Red and Red + NIR and surface soils of NIR were collected at day 15, 45, 75, and 120 using sterile petri dishes at five different locations (20 g per site) where the most dark-colored patches appeared. Five samples from each incubation were pooled. Since no BSCs formed under NIR, the top surface soils (2 mm thick) were sampled. A total of 12 samples were collected. Visual observation of appearance and measurements of soil physiochemical properties were immediately conducted, while for DNA-based analyses, the samples were stored at -80°C until further treatment.

### Characterization of BSCs and Soil Samples

The thickness of BSCs was measured with a digital caliper. Soil pH was determined on each of the replicate samples using a pH meter (HI 2221 Calibration CheckPh/ORP Meter, Hanna Instruments, United States). Briefly, 5.0 g fresh soil was mixed with 45 ml deionized water in a 100 ml beaker and then placed in a shaker for 30 min before the pH measurement.

Chlorophyll pigments was extracted in ethanol with chlorophyll concentration determined according to Lan et al. (2011). Briefly, 0.5 g sample was added into 10 ml ethanol and kept in dark for 24 h. The spectra were measured on a microplate reader (Synergy H4 Hybrid Reader, BioTek, United States) at 665 nm. Bacteriochlorophyll pigments were extracted from fresh BSCs samples with pre-cooled acetone/methanol (7:2) for 2 h. The bacteriochlorophylls fraction was analyzed on a ZORBAX SB-C18 (5 μm particle size, 150 mm × 4.6 mm) using the method modified from Chen et al. (2016). Solvents A, B, and C were 80:20 (v/v) methanol/ 0.5 M ammonium acetate, 85/15 (v/v) acetonitrile/water and ethyl acetate, respectively. Pigments were eluted at 1 mL/min at 30°C following a linear gradient of 0.0 min (100% A), 10.0 min (100% B), 27.5 min (10% B, 90% C), 29.0 min (100% B) and 31.0 min (100% B). BChl *a* was monitored by checking absorbance at 771 nm.

Available phosphorus (AP) was determined with 0.5 mol/L NaHCO_3_ (pH 8.5) (ISSCAS, 1978). Available nitrogen (AN) was determined by the alkaline diffusion method (ISSCAS, 1978). Soil organic matter (OM) was determined with the K_2_Cr_2_O_7_-H_2_SO_4_ volumetric dilution heating method (Nelson and Sommers, 1982).

### DNA Extraction, PCR Amplification and High-Throughput Sequencing

Genomic DNA was extracted from a fraction of soil sample (0.5 g) using the E.Z.N.A. Soil DNA Kit (Omega, United States) according to the manufacturer’s instructions. DNA extraction was repeated three times for each sample and the resulted DNA were pooled and stored at -20°C.

The *puf*M gene encoding for M subunit of core photosynthetic reaction centers of AAnPB was employed to quantitate the copy numbers and community structure of AAnPB (Beja et al., 2002; Yutin et al., 2005; Jiao et al., 2007; Ferrera et al., 2017; Lehours et al., 2018). PCR amplification and sequencing of *puf*M genes were conducted at the MajorBio Inc. in Shanghai, China. The PCR conditions were 95°C for 3 min, followed by 35 cycles at 95°C for 30 s, 58°C for 30 s, and 72°C for 45 s, and then a final extension step at 72°C for 10 min. Primer pairs *puf*M_557F/*puf*M_WAW (see Supplementary Table S1, Beja et al., 2002; Yutin et al., 2005) were used for partial *puf*M gene amplification. PCR was performed in a 20 μL reaction containing ca. 10 ng template DNA, 2 μL 10X buffer, 2 μL dNTPs (2.5 mM each), 0.8 μL of forward and reverse primers (5 μM each), 0.2 μL TaKaRa *rTaq* DNA polymerase. High throughput NGS sequencing was performed on an Illumina MiSeq platform (PE300) according to the standard protocols and the sequences were processed following the protocol of Xiao and Veste (2017). The MiSeq fastq files were deposited in the NCBI Sequence Read Archives (SRA) under the accession number SRP159536.

### AAnPB Assemblages

Raw *puf*M sequence data were removed of chimeras using Uchime_ref command in UPARSE v7.1^1^ (Edgar, 2013), then clustered into operational taxonomic units (OTUs) at the 94% sequence similarity, a threshold proposed by Zeng et al. (2007) based on the *puf*M sequences from pure cultures. The most abundant sequence from each OTU was selected as the representative sequence for that OTU. The OTU diversity was analyzed using QIIME v1.9.0^2^ (Caporaso et al., 2010) following the standard workflow, and the α diversity index were calculated using the mothur program v.1.30.1 (Schloss et al., 2009)^3^. Taxonomy was assigned to the OTU representative sequences (phylotypes) using BLAST (version 2.2.30+, Altschul et al., 1997; *E*-value < 10^−35^) against a custom reference collection of 378 *puf*M sequences, which was compiled from cultured or otherwise well-characterized species in GenBank.

The dissimilarity of sample assemblages was processed and plotted through Partial Least Squares Discriminant Analysis (PLS-DA) (Westerhuis et al., 2010). CoNet (Faust and Raes, 2012) was used to generate interaction network, where the OTU present in less than 4 samples were excluded. Network of significant co-occurrences was visualized using Cytoscape v.3.5.1 (Shannon et al., 2003). Significant positive and negative correlations between OTUs were determined individually by support of Pearson’s correlation measures. Networks from the measures on samples of three illumination treatments (Red, NIR, and Red + NIR) were merged by intersection, keeping only significant interactions (α set at 0.05) with support from all methods.

### Quantification of Gene Copy Numbers

Quantitative real-time PCR (qPCR) was used to quantify copies of bacterial 16S rRNA genes, AAnPB’s *puf*M genes, diazotrophs’ *nif*H genes, fungal 18S rRNA genes, cyanobacterial 16S rRNA genes and eukaryotic microalgal 18S rRNA genes using specific primers and temperature profiles (listed in Supplementary Table S1). Briefly, the target gene was PCR amplified in a 20 μL reaction mixture containing 10 μL SYBR^®^ *Premix Ex Taq* Kit (TaKaRa), 0.5 μM of each primer, and 5–10 ng template DNA. The target DNA fragments were amplified and cloned into the pEASY-T1 vector (TransGen, Beijing, China), then the recombinant plasmid was further confirmed by Sanger sequencing using the universal primers for plasmid. Quantification of the recombinant plasmid was performed using a NanoDrop 2000 (Thermo Fisher Scientific, Wilmington, DE, United States). Standard curve for each gene was generated by a 10-fold dilution series of the recombinant plasmid DNA. The qPCR was performed in triplicate, and the specificity of amplification was confirmed by agarose gel electrophoresis and melting curve analysis. The primers were synthesized by Sangon Biological Engineering Technology and Service Co. (Shanghai, China), and the quantification PCR was performed on a Roche LightCycler 480II platform.

### Effect of NIR Radiation on the Growth of Cyanobacteria

To test whether NIR light alone could support the growth of chlorophylls-harboring photoautotrophs, a cyanobacterial strain MDM25 was incubated under NIR light in BG11 liquid medium (50 mL, pH 7.5) in a 100 mL flask with shaking every 6 h. The strain MDM25 was isolated by our lab from BSCs of the Desert Hopq and tentatively classified as *Microcoleus vaginatus* based on its 16S rRNA gene sequence (GenBank Accession No. MH853840), which is a common pioneer species occurring in BSCs (Belnap et al., 2016). After 30 days of incubation, the light absorption spectra were determined and the OD_600_ values were compared to demonstrate whether any growth occurs.

### Statistical Analyses

All statistical analyses were done with the software IBM SPSS Amos 19.0. The liner regression analysis were done with GraphPad Prism 7.0 using the Pearson’s correlation coefficient. The significant difference was calculated using Tukey’s test under *P* < 0.05.

## Results

### Monitoring of the Development of Biological Soil Crusts and the Physiochemical Properties of Soils

**FIGURE 2:**
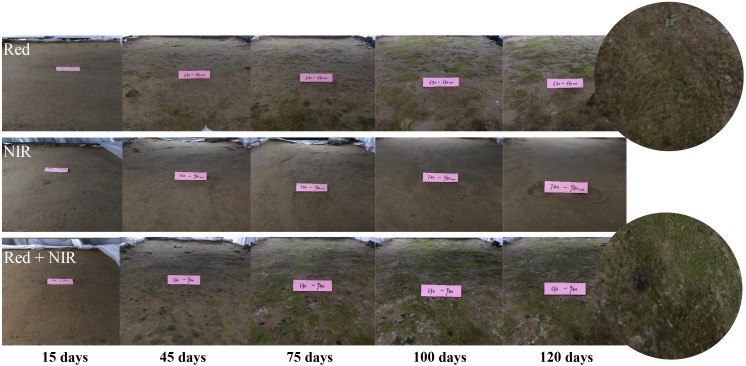
Visible changes on the surface soils incubated under three different types of light sources. The digital photographs showed the apparent changes of biological soil crusts (BSCs) at day 15, 45, 75, 100, and 120. Red: red light; NIR, near-infrared light.

Under near infrared light (NIR), no BSCs formed over the entire period, while BSCs were well developed under Red or Red + NIR (Figure 2). Very thin layer of BSCs began to appear under Red roughly at day 15, while under Red + NIR, BSCs firstly appeared at day 6. After the first appearance, the crusts of Red + NIR developed more rapidly than that of Red. The earliest crusts were dominated by greenish cyanobacteria and then moss gradually appeared. In the crusts of Red + NIR, moss appeared earlier and their coverage was denser than those of Red alone. Moreover, with the increase of NIR intensity, the development of BSCs was obviously accelerated (Supplementary Figure S1).

In addition to the visible changes in the appearance of BSCs, changes in soil physiochemical properties occurred at both top soils and subsoils, e.g., pH decreased and nutrient levels (as reflected by AN, AP, OM) increased along with incubation time (Table 1 and Supplementary Table S2). By the end of experiment, the Red + NIR crusts was 23.97% thicker than the Red crusts. Higher concentrations of chlorophyll and OM were also observed in Red + NIR, up to 1.8 and 2.3 times of those in Red, respectively. Under Red + NIR, AP was only slightly higher than that of Red, while AN was even less than that of Red, which may be caused by a high nutrient consumption by the bacteria, fungi and moss communities. Interestingly, although sole NIR illumination did not lead to the formation of BSCs, it did enhance the soil’s nutrient level (e.g., top soil at days 15 and 120, OM from 0.04 mg/kg increased to 1.37 mg/kg, AP from 2.34 mg/kg to 17.85 mg/kg, AN from 12.66 mg/kg to 23.80 mg/kg), yet no chlorophyll or bacteriochlorophyll was detected. These results indicated that the combination of red and near-infrared lights remarkably promoted the development of BSCs.

**Table 1:** Physiochemical properties of biological soil crusts (Red and Red + NIR) and surface soil (NIR) at day 120.

Samples	pH	AP (mg⋅kg^−1^)	AN (mg⋅kg^−1^)	OM (g⋅kg^−1^)	*Chl a* (μg⋅kg^−1^)	*BChl a* (ng⋅g^−1^)	Thickness (mm)	Density (g⋅cm^−3^)
Red	7.67 ± 0.03^a^	19.05 ± 0.06^b^	43.63 ± 1.01^a^	2.98 ± 0.09^b^	15.10 ± 0.20^b^	256.37 ± 3.58^b^	10.51 ± 0.43^b^	1.54 ± 0.05^a^
NIR	7.60 ± 0.01^b^	17.85 ± 0.23^c^	23.80 ± 1.75^c^	1.37 ± 0.17^c^	*N.D.*	*N.D.*	*N.D.*	*N.D.*
Red + NIR	7.47 ± 0.02^c^	21.38 ± 0.25^a^	40.72 ± 1.01^b^	6.07 ± 0.09^a^	17.90 ± 0.24^a^	277.73 ± 3.14^a^	13.03 ± 0.56^a^	1.60 ± 0.06^a^

*Data are means ± standard error. Letters (a, b, or c) in superscript represent the observed significant differences (Turkey’s test; P < 0.05). AN, available nitrogen; AP, available phosphorous; OM, organic matter; Chl a, Chlorophyll a; BChl a, bacteriochlorophyll a; N.D., not detected. Thickness and density were measured for crusts.*

### Abundance of Bacteria, AAnPB, Diazotrophs, Fungi, Cyanobacteria and Microalgae

**FIGURE 3:**
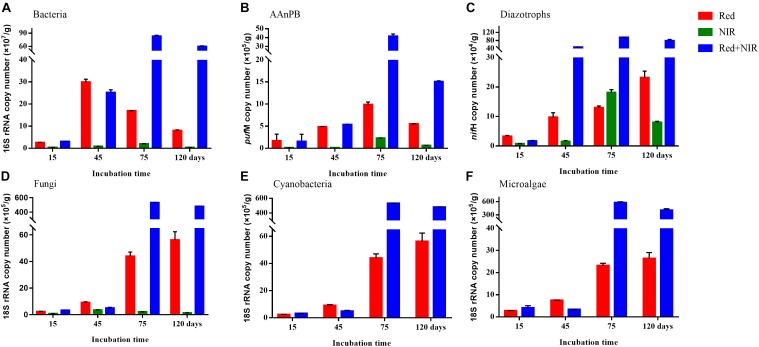
Copy numbers (mean ± standard error) of total bacteria **(A)**, AAnPB **(B)**, diazotrophs **(C)**, fungi **(D)**, cyanobacteria **(E),** and microalgae **(F)** in biological soil crusts and surface soils per gram sample under Red, NIR and Red + NIR illumination respectively. AAnPB, aerobic anoxygenic phototrophic bacteria.

**FIGURE 4:**
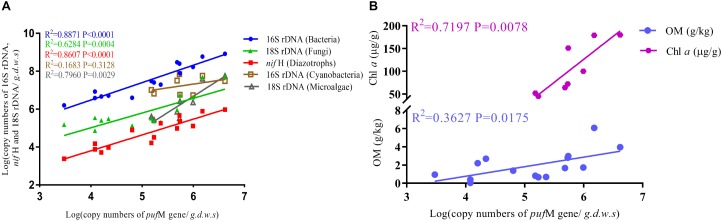
Correlation of the absolute abundance of AAnPB with the abundance of cyanobacteria, microalgae, total bacteria, fungi and diazotrophs **(A)**, and with soil characteristics (Chl *a*, OM) **(B)**.

The 16S rRNA, 18S rRNA, *puf*M and *nif*H genes all showed a significant increase in copy numbers along incubation time and most of genes reached their highest copy numbers at day 75 (Figure 3 and Supplementary Table S3). The largest increment was ca. 5.3 × 10^6^ for fungi and microalgae under Red + NIR from day 15 to day 75. For individual genes, there were large differences between exposures to different illuminations. However, Red + NIR always showed the highest gene copy numbers, indicating that near-infrared could stimulate the growth of AAnPB, which might further promote the growth of other microbes. Under NIR, no cyanobacteria or microalgae were found, and other groups were 1–2 orders of magnitude less than those of Red or Red + NIR. The regression analysis revealed that the abundance of AAnPB was significantly and positively correlated with the concentrations of chlorophyll *a* (*r* = 0.6022, *P* < 0.05) and organic matter (*r* = 0.8483, *P* < 0.01), and with the abundance of microalgae (*r* = 0.8922, *P* < 0.01), total bacteria (*r* = 0.9419, *P* < 0.01), fungi (*r* = 0.7927, *P* < 0.01) and diazotrophs (*r* = 0.9278, *P* < 0.01), but not with cyanobacteria (Figure 4).

### Genetic Diversity and Composition of AAnPB Communities

**FIGURE 5:**
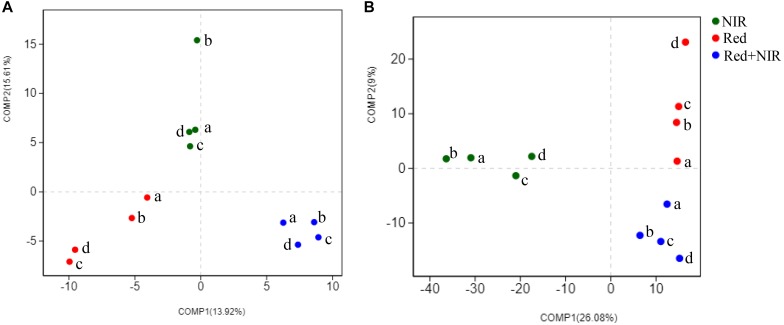
Partial Least Squares Discriminant Analysis of AAnPB **(A)** and total bacteria **(B)** at the OTU level for distinguishing the intergroup community structure difference. a, b, c, and d mean the cultivation days of 15, 45, 75, 120 under three illumination treatments, respectively.

High-throughput sequencing of *puf*M gene was used to reveal the community structure of AAnPB. After the quality control of sequences, a total of 689,063 sequences (average length of 264 bp) were obtained. The *puf*M gene sequences were grouped into 337 OTUs at the sequence identity cutoff of 94% (Supplementary Table S4). All coverage values were over 99%, suggesting the sequencing depth was sufficiently high to represent the real diversity and composition of AAnPB communities. The PLS-DA analysis based on OTU level demonstrated that the AAnPB species clustered into three separate groups corresponding to three types of light source, i.e., Red, NIR and Red + NIR (Figure 5), suggesting that different light sources greatly affected the community structures of AAnPB. At the early times (day 15 and 45), the diversity indices (Ace and Chao1) (Supplementary Table S4) of Red was slightly higher than those of Red + NIR. However, at day 75, Red and Red + NIR were similar to each other in terms of AAnPB diversity level, while the Red + NIR showed a slightly higher diversity than that of Red at day 120. And, the Ace and Chao1 values of NIR were always much lower than that of Red or Red + NIR. Indices of Shannon followed a similar pattern. The comparison results of AAnPB diversity under different light regimes suggested that although the AAnPB diversity under NIR alone was not high, the near-infrared radiations could indeed lead to an increased diversity of AAnPB in BSCs when used in combination with red lights.

**FIGURE 6:**
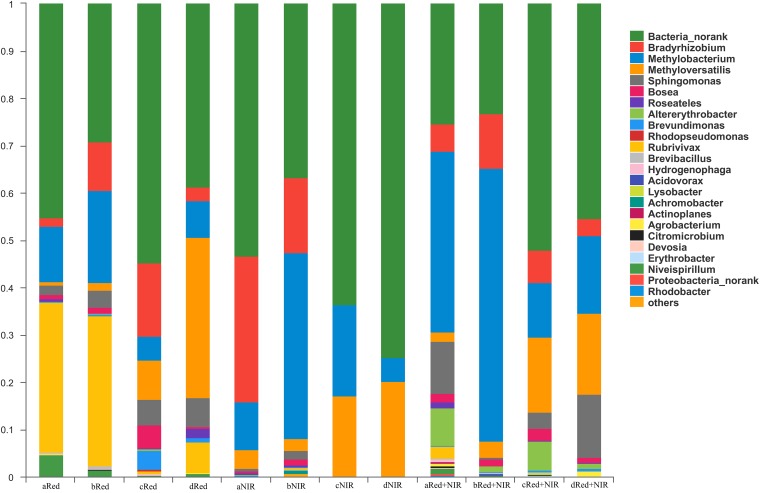
Community structure and composition of aerobic anoxygenic phototrophic bacteria (AAnPB) in the biological soil crusts of Red and Red + NIR and in the surface soils of NIR (at the level of genus). Unknown genera are designated as Bacteria_norank. Groups of less than 0.1%, including *Gemmatimonadetes*, were classified as others. In the sample names (e.g., aRed, bRed, cRed, dRed, and etc.), a, b, c, and d means the cultivated days of 15, 45, 75, 120, respectively

AAnPB communities were dominated by the unknown group designated as Bacteria_norank and members of phylum Proteobacteria, including classes Alphaproteobacteria and Betaproteobacteria, which include the genera *Bradyrhizobium*, *Methylobacterium*, *Methyloversatilis*, *Sphingomonas*, *Bosea*, *Roseatles*, *Altererythrobacter*, *Brevundimonas*, *Rubrivivax*, *Niveispirillum*, and *Rhizobium*. At the genus level, the composition of AAnPB communities under different illuminations were very different from each other (Figure 6). When exposed to NIR or Red + NIR, the fraction of unknown taxon Bacteria_norank gradually increased and became the absolutely dominant group, esp. at day 75 and day 120. This suggests that many unknown AAnPB exist in desert soils and near-infrared radiations could be a potential enriching means for AAnPB. In contrast to NIR or Red + NIR, the genera *Rubrivivax* and *Niveispirillum* constituted a much higher proportion of AAnPB community under Red.

The Pearson’s correlation analysis showed that *Bradyrhizobium*, *Sphingomonas*, unknown *Gemmatimonadetes* and unclassified Proteobacteria were significantly (*P* < 0.05) correlated with OM. The similar strong correlations were observed between chlorophyll *a*, AN and OM and *Bradyrhizobium*, *Methyloversatilis*, *Skermanella* and unclassified Proteobacteria, and between *Methyloversatilis* and pH, AN, and AP (Supplementary Table S5). These strong correlations suggest that AAnPB were actively involved in the nutrient cycle within BSCs.

**FIGURE 7:**
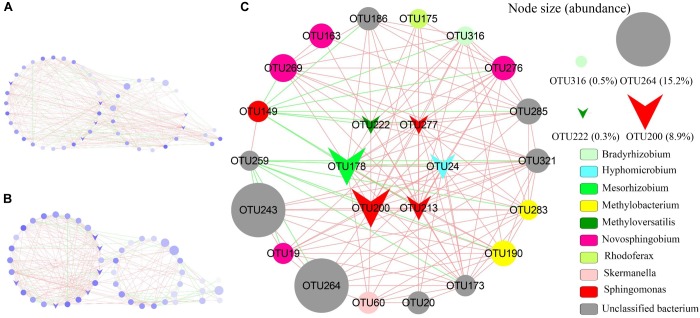
Network of phylotypes of aerobic anoxygenic phototrophic bacteria based on *puf*M sequences present in biological soil crusts of Red + NIR illumination with significant pairwise correlations. **(A)** Global network with three modules of α-, β-, γ-Proteobacteria (left to right); **(B)** global network with three modules showed directly, indirectly and non-connected to putative keystones (left to right); **(C)** detailed sub-network showed direct connection to putative keystone species. Circles indicate individual OTUs; circle diameter represents the relative abundance; triangle symbols are putative keystones; red and green edges indicate co-occurrence (positive interaction) and mutual-exclusion (negative interaction). See Supplementary Table S6 for the taxonomic affiliations of OTUs.

Co-occurrence networks (CoNet) analysis was further conducted to demonstrate the interactions among AAnPB species in BSCs of Red + NIR. In CoNet analysis, the phylotypes that are significantly (*P* < 0.05) linked are often referred to as the “keystone species” or hub playing important roles (Wang et al., 2017). Keystone taxa have also been frequently referred to as “ecosystem engineers” owing to their large influence in the community (Dunne et al., 2002). High mean degree, high closeness centrality and low betweenness centrality can be collectively used to identify keystone taxa with 85% accuracy (Berry and Widder, 2014). Here we observed that there were three intra-connected modules (Figure 7). Six OTUs were closely related to *Mesorhizobium* (OTU178), *Methyloversatilis* (OTU222), *Hydrogenophaga* (OTU24), and *Sphingomonas* (OTU200, OTU213, and OTU277), where centrality degree were over 0.5 and 94% of total linkages were positive interactions (Supplementary Table S6).

### Effect of Lights at Different Wavelengths on the Growth of Cyanobacteria

Over a long period of time (30 days) of incubation for *Microcoleus vaginatus* MDM25, there was no growth observed under NIR alone, whereas apparent growth (cultures turned from colorless to dark greenish) occurred under the Red or Red + NIR setting. No significant difference in the biomass of *M. vaginatus* in terms of the value of OD_600_ or chlorophyll concentrations was observed between Red and Red + NIR (Supplementary Figure S2). Since *M. vaginatus* was a photoautotrophic representative of cyanobacteria isolated from BSCs, it is very likely that the whole chlorophylls-based photoautotrophic community in natural BSCs do not respond to near IR irradiations either, and thus the changes in BSCs triggered by NIR in our experiments were most likely caused by the NIR-absorbing AAnPB instead of chlorophylls-containing photoautotrophs.

## Discussion

In BSCs, photoautotrophs like cyanobacteria and algae attracted extensive attention, owing to their abilities and contributions related to nutrition enhancement (e.g., carbon and nitrogen fixation), the binding of sand particles through secreted polysaccharides or filamentous cells (Hallenbeck, 2017). Whether the widespread AAnPB in natural ecosystems (Madigan, 2003; Koblížek, 2015) play a role in the development of BSCs has not been addressed so far. In this study, we took advantage of the different light absorption properties in chlorophyll-containing photoautotrophs (trap red light but not utilize NIR) and BChl *a*-containing AAnPB (trap NIR but not utilize red light) (Kolber et al., 2001) and set up a microcosm system provided with different light conditions (Red vs. NIR vs. Red + NIR). Although the system did not fully mimic the environmental factors in desert, e.g., hard light, wind blow, drought stress and diurnal temperature variations, we did observe the strong effects exerted by NIR on the development of BSCs, which could be attributed to the activities of microbial seeds in BSCs.

### Near IR Radiations Stimulate the Growth of AAnPB in BSCs

Biological soil crusts only formed in Red and Red + NIR treatments, but not in NIR. However, BSCs of Red + NIR were significantly thicker and denser than those of Red (Figure 2 and Table 1), which indicated that the combined illumination of red light and NIR led to a much better development of BSCs than red light or NIR illumination alone. Moreover, the obvious acceleration of the development of BSCs with the increase of NIR intensity further confirmed the role of NIR and AAnPB in the development of BSCs (Figure 2). Our batch culture experiments showed that there was no difference between the treatment of Red and Red + NIR on our cyanobacterial cultures, and the sole NIR radiations did not allow the tested cyanobacterium to grow (Supplementary Figure S2). Since only one cyanobacterial strain was tested in this study, we could not rule out the possibility that other cyanobacterial species may respond to NIR differently or even there may exist unknown NIR absorbing cyanobacteria in natural BSCs, like the far-red absorbing (up to 706 nm by chlorophyll *f*) cyanobacterium that existed within stromatolites in an Australian bay (Chen et al., 2010). However, we did not observe any chlorophyll *f* peak on the HPLC elution profiles of our BSCs samples. To our knowledge, it is very likely that the enhanced development of BSCs after the addition of NIR radiations to red light was caused by the BChl *a*-containing AAnPB that can absorb NIR effectively.

Despite no BChl *a* was detected in natural BSCs (Nagy et al., 2005), the oxygen- and light-rich BSCs might be a highly suitable niche for AAnPB. The existence of AAnPB in BSCs has been confirmed by a culture-dependent study (Csotonyi et al., 2010), yet the contribution of BChl-containing phototrophs to the development of BSCs has long been overlooked because BChl *a* was not detected. The undetectable concentration of BChl *a* in BSCs may be attributed to the typically low production of BChl *a* in AAnPB (Yurkov and Csotonyi, 2009), and in some cases the major light-harvesting pigment is spheroidenone rather than BChl *a* (Sato-Takabe et al., 2014). Furthermore, BChl *a* is sensitive to environmental factors such as strong light or UV, which can inhibit its biosynthesis and even cause the breakdown of BChl *a* (Raser et al., 1992). Since the desert is constantly lacking moisture and the sampled BSCs were generally dry or air-dried, the BChl *a* pigment in BSCs may have been subject to significant degradation and thus was not detectable.

In contrast, under laboratory conditions, we observed a high concentration of BChl *a* in BSCs of Red and Red + NIR (256.37 and 277.73 μg/g, respectively) after a 120-day incubation. Compared to environmental parameters in natural desert BSCs, the major difference in our settings was the introduction of high humidity (60 – 70%), which enabled us to observe the light effects on BSC development in a relatively short period. This might not reflect how microbial communities in BSCs develop over time in nature. However, BSCs are well known to develop extremely slowly in deserts, often taking decades to form a functional but fragile community (Belnap, 2003). We could hardly mimic such a harsh environment in a microcosm setting. Nonetheless, the microbial seeds inside the sampled BSCs were the same as in original desert BSCs. The observed increase in BChl *a* concentration in our experiment was certainly ascribed to the growth of AAnPB seeds.

Near-infrared has been demonstrated to be able to stimulate the growth of a purple bacterial strain of *Rhodopseudomonas* sp., which led to an improved efficiency of wastewater treatment process (Qi et al., 2017). Certain anaerobic green sulfur photosynthetic bacteria could even grow under monochromatic NIR light at a very low photo flux (<10 μmol.m^−2^.s^−1^, Saikin et al., 2014). In this study, we employed a NIR light intensity of 70 lux (about 7 μmol.m^−2^.s^−1^), which led to a 9 days earlier development of BSCs in the Red + NIR treatment than that in the Red alone. When the NIR intensity was increased to 140 lux, the acceleration was even more remarkable. Although there is no report that NIR alone can support the growth of AAnPB, light may improve their growth rates by increasing their utilization efficiency of organic matter (Soora and Cypionka, 2013) or enhance their capability to cope with starvation (Zhang et al., 2015).

### Ecological Significance of AAnPB in BSCs

Biological crusts are vital in creating and maintaining fertility in barren desert soils (Belnap, 2003). To protect and maintain BSCs, we first need to understand the biological processes underpinning the development of BSCs, which involves diverse microorganisms. In our study, we observed that when provided with NIR radiations in addition to Red, the total abundances of bacteria, AAnPB, diazotrophs, fungi, cyanobacteria and microalgae were greatly increased compared to Red alone, and the abundance of AAnPB was positively and remarkably correlated with those of other groups of bacteria. Since AAnPB were the only known group of these microorganisms in BSCs that could harvest the light energy in the NIR spectra, our results strongly suggested that a positive interaction exists between AAnPB and other microbes, esp. photoautotrophs like cyanobacteria. AAnPB may even play a role of stimulating the growth of other microbes and further promote the development of BSCs.

Surprisingly, despite that no BSCs were observed and no 16S rRNA genes of Cyanobacteria or Chl *a* were detected, nutrient levels in surface soils of NIR (as indicated by AP, AN and OM) were enhanced over time. The content of OM in Red + NIR could even be 2.3-fold higher than that in Red. It is unclear as to how AAnPB contribute to the large increase in the content of organic matter. One explanation could be that AAnPB promote carbon accumulation in BSCs by directly fixing carbon, in addition to the harvesting of solar energy for a reduced carbon consumption (Kolber et al., 2001). One representative species of marine AAnPB *Dinoroseobacter shibae* has been found to be able to use the ethylmalonyl-CoA pathway to fix CO_2_, and thus reduce the respiration in light when transitioning from a heterotrophic to a photoheterotrophic lifestyle (Bill et al., 2017). A recent environmental genomics survey even suggested the presence of a widespread group of AAnPB in global oceans that could potentially fix CO_2_ under aerobic conditions through RubisCO (Graham et al., 2018). It would be interesting to further research whether AAnPB have contributed to the carbon accumulation in BSCs in a similar manner. On the other hand, some AAnPB, e.g., *Methylobacterium*, *Bradyrhizobium*, and *Methyloversatilis*, could fix air nitrogen and/or make insoluble phosphorus compounds solubilized (Yurkov and Csotonyi, 2009; Chauhan et al., 2015; Good et al., 2015). These groups abounded in one period or the whole time in the incubation (Figure 6) and total AAnPB were strongly correlated to nutrients (AP, AN, OM) level (Supplementary Table S5). These supported that AAnPB were involved in some major nutrient cycles within BSCs.

If we assume an average of 5 copies of 16S rRNA gene per bacteria cell and one *puf*M copy per AAnPB cell, the proportion of AAnPB to total bacteria in our simulated BSCs is 0.81 – 3.45%, 0.71 – 8% and 1.26 – 2.49% in Red, NIR and Red + NIR, respectively. A similar percentage (0.1 – 5.9%) was reported by Csotonyi et al. (2010) in a cultivation-dependent survey of AAnPB in three Canadian soil crust communities, where the dominate AAnPB species were *Methylobacterium* and *Sphingomonas*. Similarly, the AAnPB communities in our samples were dominated by *Methyloversatilis* and *Sphingomonas* as revealed by high-throughput sequencing of *puf*M genes, suggesting that these two genera may represent common keystone species in the AAnPB communities of BSCs.

Indeed, methylotrophs (e.g., *Methyloversatilis*) and *Sphingomonas* were located in the centrality in the AAnPB network graphs and were positively connected with other nodes even without any negative connection. Network graph, through which the co-existence interaction could be explored, is a powerful tool to predict the key species in communities (Shi et al., 2016; Van Goethem et al., 2017). An example was that Cyanobacteria and Alphaproteobacteria were identified as keystone species in the hypolithic BSCs through a co-occurrence network analysis (Van Goethem et al., 2017). Here we also observed that positive relationships predominated in the interactions of the AAnPB communities, in agreement with that positive co-occurrence often dominated in extreme environments like deserts (Fierer et al., 2012; Van Goethem et al., 2017).

Members of *Methyloversatilis* could utilize diverse methyl compounds (Good et al., 2015) and even release NH_3_ for the benefits of other microbes when methylamine was dissimilated (Taubert et al., 2017). *Sphingomonas* could use diverse compounds and secret polysaccharides (Li et al., 2017), as cyanobacteria often do during the development of BSCs. These species appear to be able to facilitate specialized soil processes that are related to carbon cycling and thus might impose larger impacts on carbon dynamics than other non-keystone species. Future isolation of pure cultures of these keystone species from desert BSCs can help to answer how they facilitate the development of BSCs.

In summary, the addition of a small intensity of near-infrared light did not lead to an apparent increase in total intensity of light, but caused a remarkable promotion on the development of BSCs in our microcosms. Near-infrared light may function as a complementary energy source to organic matter for AAnPB and make them more competitive over obligate heterotrophic bacteria in nutrient poor environments like desert soils. Such promotion of BSCs may occur via the enhancement of soil nutrition and the positive interaction between photoautotrophs and some key AAnPB species, among which methylotrophs and *Sphingomonas* members are the major contributors. Overall, this study provides the first insight into the role of AAnPB in the development of BSCs and may open a new prospect for understanding the eco-physiological functions of AAnPB in fragile dryland ecosystems.

## Author Contributions

KT carried out the experiments and prepared the draft of the manuscript. KT, FF, and YZ analyzed the data. LJ, BY, SY, HL, and JM helped with the deserts samples and prepared the experiments. FF and YZ designed the research and wrote the manuscript with input from KT.

## Conflict of Interest Statement

The authors declare that the research was conducted in the absence of any commercial or financial relationships that could be construed as a potential conflict of interest.

## Footnotes

1 http://drive5.com/uparse/

2 http://qiime.org/

3 http://www.mothur.org/wiki/Schloss_SOP#Alpha_diversity

## References

[B1] Altschul S. F. Madden T. L. Schäffer A. A. Zhang J. Zhang Z. Miller W. (1997). Gapped BLAST and PSI-BLAST: a new generation of protein database search programs. *Nucleic Acids Res.* 25 3389–3402. 10.1093/nar/25.17.3389 9254694 PMC146917

[B2] Beja O. Suzuki M. T. Heidelberg J. F. Nelson W. C. Preston C. M. Hamada T. (2002). Unsuspected diversity among marine aerobic anoxygenic phototrophs. *Nature* 415 630–633. 10.1038/415630a 11832943

[B3] Belnap J. (2003). The world at your feet: desert biological soil crusts. *Front. Ecol. Environ.* 1:181–189. 10.2307/3868062

[B4] Belnap J. Weber B. Büdel B. (2016). “Biological soil crusts as an organizing principle in drylands,” in *Biological Soil Crusts: An Organizing Principle in Drylands*, eds Weber B. Büdel B. Belnap J. (Cham: Springer), 3–13. 10.1007/978-3-319-30214-0_1

[B5] Berry D. Widder S. (2014). Deciphering microbial interactions and detecting keystone species with co-occurrence networks. *Front. Microbiol.* 5:219. 10.3389/fmicb.2014.00219 24904535 PMC4033041

[B6] Bill N. Tomasch J. Riemer A. Müller K. Kleist S. Schmidt-Hohagen K. (2017). Fixation of CO2 using the ethylmalonyl-CoA pathway in the photoheterotrophic marine bacterium *Dinoroseobacter shibae*. *Environ. Microbiol.* 19 2645–2660. 10.1111/1462-2920.13746 28371065

[B7] Caporaso J. G. Kuczynski J. Stombaugh J. Bittinger K. Bushman F. D. Costello E. K. (2010). QIIME allows analysis of high-throughput community sequencing data. *Nat. Methods* 7 335–336. 10.1038/nmeth.f.303 20383131 PMC3156573

[B8] Chauhan H. Bagyaraj D. J. Selvakumar G. Sundaram S. P. (2015). Novel plant growth promoting rhizobacteria–prospects and potential. *Appl. Soil Ecol.* 95 38–53. 10.1016/j.apsoil.2015.05.011

[B9] Chen G. E. Canniffe D. P. Martin E. C. Hunter C. N. (2016). Absence of the cbb3 terminal oxidase reveals an active oxygen-dependent cyclase involved in bacteriochlorophyll biosynthesis in *Rhodobacter sphaeroides*. *J. Bacteriol.* 198 2056–2063. 10.1128/JB.00121-16 27215788 PMC4944227

[B10] Chen M. Schliep M. Willows R. D. Cai Z. L. Neilan B. A. Scheer H. (2010). A red-shifted chlorophyll. *Science* 329 1318–1319. 10.1126/science.1191127 20724585

[B11] Chew A. G. Bryant D. A. (2007). Chlorophyll biosynthesis in bacteria: the origins of structural and functional diversity. *Annu. Rev. Microbiol.* 61 113–129. 10.1146/annurev.micro.61.080706.093242 17506685

[B12] Csotonyi J. T. Swiderski J. Stackebrandt E. Yurkov V. (2010). A new environment for aerobic anoxygenic phototrophic bacteria: biological soil crusts. *Environ. Microbiol. Rep.* 2 651–656. 10.1111/j.1758-2229.2010.00151.x 23766251

[B13] Dunne J. A. Williams R. J. Martinez N. D. (2002). Network structure and biodiversity loss in food webs: robustness increases with connectance. *Ecol. Lett.* 5 558–567. 10.1046/j.1461-0248.2002.00354.x 23776404

[B14] Edgar R. C. (2013). UPARSE: highly accurate OTU sequences from microbial amplicon reads. *Nat. Methods* 10 996–998. 10.1038/nmeth.2604 23955772

[B15] Elbert W. Weber B. Burrows S. Steinkamp J. Büdel B. Andreae M. O. (2012). Contribution of cryptogrammic covers to the global cycles of carbon and nitrogen. *Nat. Geosci.* 5 459–462. 10.1038/ngeo1486

[B16] Faust K. Raes J. (2012). Microbial interactions: from networks to models. *Nat. Rev. Microbiol.* 10 538–550. 10.1038/nrmicro2832 22796884

[B17] Feng S. Fu Q. (2013). Expansion of global drylands under a warming climate. *Atmos. Chem. Phys.* 13 10081–10094. 10.5194/acpd-13-14637-2013 26591879

[B18] Ferrera I. Sarmento H. Priscu J. C. Chiuchiolo A. González J. M. Grossart H. P. (2017). Diversity and distribution of freshwater aerobic anoxygenic phototrophic bacteria across a wide latitudinal gradient. *Front. Microbiol.* 8:175. 10.3389/fmicb.2017.00175 28275369 PMC5320280

[B19] Fierer N. Leff J. W. Adams B. J. Nielsen U. N. Bates S. T. Lauber C. L. (2012). Cross-biome metagenomic analyses of soil microbial communities and their functional attributes. *Proc. Natl. Acad. Sci. U.S.A.* 109 21390–21395. 10.1073/pnas.1215210110 23236140 PMC3535587

[B20] Good N. M. Lamb A. Beck D. A. Martinez-Gomez N. C. Kalyuzhnaya M. G. (2015). C1-pathways in *Methyloversatilis universalis* FAM5: genome wide gene expression and mutagenesis studies. *Microorganisms* 3 175–197. 10.3390/microorganisms3020175 27682085 PMC5023235

[B21] Graham E. D. Heidelberg J. F. Tully B. J. (2018). Potential for primary productivity in a globally-distributed bacterial phototroph. *ISME J.* 12 1861–1866. 10.1038/s41396-018-0091-3 29523891 PMC6018677

[B22] Hallenbeck P. C. (2017). *Modern Topics in the Phototrophic Prokaryotes: Environmental and Applied Aspects*. Cham: Springer 10.1007/978-3-319-46261-5

[B23] Hatzianastassiou N. Matsoukas C. Fotiadi A. Pavlakis K. G. Drakakis E. Hatzidimitriou D. (2005). Global distribution of Earth’s surface shortwave radiation budget. *Atmos. Chem. Phys.* 5 2847–2867. 10.5194/acp-5-2847-2005

[B24] ISSCAS (1978). *Institute of Soil Science, Chinese Academy Science. Physical and Chemical Analysis Methods of Soils*. Shanghai: Shanghai Science Technology Press.

[B25] Jiao N. Zhang Y. Zeng Y. Hong N. Liu R. Chen F. (2007). Distinct distribution pattern of abundance and diversity of aerobic anoxygenic phototrophic bacteria in the global ocean. *Environ. Microbiol.* 9 3091–3099. 10.1111/j.1462-2920.2007.01419.x 17991036

[B26] Koblížek M. (2015). Ecology of aerobic anoxygenic phototrophs in aquatic environments. *FEMS Microbiol. Rev.* 39 854–870. 10.1093/femsre/fuv032 26139241

[B27] Kolber Z. S. Plumley F. G. Lang A. S. Beatty J. T. Blankenship R. E. VanDover C. L. (2001). Contribution of aerobic photoheterotrophic bacteria to the carbon cycle in the ocean. *Science* 292 2492–2495. 10.1126/science.1059707 11431568

[B28] Lan S. Wu L. Zhang D. Hu C. Liu Y. (2011). Ethanol outperforms multiple solvents in the extraction of chlorophyll-a from biological soil crusts. *Soil Biol. Biochem.* 43 857–861. 10.1016/j.soilbio.2010.12.007

[B29] Lehours A. C. Enault F. Boeuf D. Jeanthon C. (2018). Biogeographic patterns of aerobic anoxygenic phototrophic bacteria reveal an ecological consistency of phylogenetic clades in different oceanic biomes. *Sci. Rep.* 8:4105. 10.1038/s41598-018-22413-7 29515205 PMC5841314

[B30] Li Y. Xu L. Gong H. Ding B. Dong M. Li Y. (2017). A microbial exopolysaccharide produced by *Sphingomonas* species for enhanced heavy oil recovery at high temperature and high salinity. *Energy Fuels* 31 3960–3969. 10.1021/acs.energyfuels.6b02923

[B31] Madigan M. T. (2003). Anoxygenic phototrophic bacteria from extreme environments. *Photosynth. Res.* 76 157–171. 10.1023/A:1024998212684 16228575

[B32] Nagy M. L. Pérez A. Garcia-Pichel F. (2005). The prokaryotic diversity of biological soil crusts in the sonoran desert (organ pipe cactus national monument. AZ). *FEMS Microbiol. Ecol.* 54 233–245. 10.1016/j.femsec.2005.03.011 16332322

[B33] Nelson D. W. Sommers L. E. (1982). “Total carbon, organic carbon and organic matter,” in *Methods of Soil Analysis, Part 2. Chemical and Microbiological Properties*, eds Page A. L. Miller R. H. Keeney D. R. (Madison, WI: SSSA), 539–577.

[B34] Porada P. Weber B. Elbert W. Pöschl U. Kleidon A. (2013). Estimating global carbon uptake by lichens and bryophytes with a process-based model. *Biogeosciences* 10 6989–6989. 10.5194/bg-10-6989-2013

[B35] Porada P. Weber B. Elbert W. Pöschl U. Kleidon A. (2014). Estimating impacts of lichens and bryophytes on global biogeochemical cycles. *Glob. Biogeochem. Cycles* 28 71–85. 10.1002/2013GB004705

[B36] Qi X. Ren Y. Tian E. Wang X. (2017). The exploration of monochromatic near-infrared LED improved anoxygenic photosynthetic bacteria Rhodopseudomonas sp. for wastewater treatment. *Bioresour. Technol.* 241 620–626. 10.1016/j.biortech.2017.05.202 28605726

[B37] Raser L. N. Thomas L. L. Kim J. H. Cotton T. M. Uphaus R. A. (1992). Factors determining stability of bacteriochlorophyll monolayers. *Thin Solid Films* 210 753–755. 10.1016/0040-6090(92)90394-Q

[B38] Saikin S. K. Khin Y. Huh J. Hannout M. Wang Y. Zare F. (2014). Chromatic acclimation and population dynamics of green sulfur bacteria grown with spectrally tailored light. *Sci. Rep.* 4:5057. 10.1038/srep05057 24862580 PMC4033924

[B39] Sato-Takabe Y. Hamasaki K. Suzuki K. (2014). Photosynthetic competence of the marine aerobic anoxygenic phototrophic bacterium *Roseobacter* sp. under organic substrate limitation. *Microbes Environ.* 29 100–103. 10.1264/jsme2.ME13130 24492676 PMC4041232

[B40] Schloss P. D. Westcott S. L. Ryabin T. Hall J. R. Hartmann M. Hollister E. B. (2009). Introducing mothur: open-source, platform-independent, community-supported software for describing and comparing microbial communities. *Appl. Environ. Microbiol.* 75 7537–7541. 10.1128/AEM.01541-09 19801464 PMC2786419

[B41] Shannon P. Markiel A. Ozier O. Baliga N. S. Wang J. T. Ramage D. (2003). Cytoscape: a software environment for integrated models of biomolecular interaction networks. *Genome Res.* 13 2498–2504. 10.1101/gr.1239303 14597658 PMC403769

[B42] Shi S. Nuccio E. E. Shi Z. J. He Z. Zhou J. Firestone M. K. (2016). The interconnected rhizosphere: high network complexity dominates rhizosphere assemblages. *Ecol. Lett.* 19 926–936. 10.1111/ele.12630 27264635

[B43] Soora M. Cypionka H. (2013). Light enhances survival of *Dinoroseobacter shibae* during long-term starvation. *PLoS One* 8:e83960. 10.1371/journal.pone.0083960 24386315 PMC3875502

[B44] Stomp M. Huisman J. Stal L. J. Matthijs H. C. (2007). Colorful niches of phototrophic microorganisms shaped by vibrations of the water molecule. *ISME J.* 1 271–282. 10.3389/fmicb.2012.00298 18043638

[B45] Tahon G. Tytgat B. Stragier P. Willems A. (2016). Analysis of cbbL, nifH, and pufLM in soils from the sør rondane mountains, antarctica, reveals a large diversity of autotrophic and phototrophic bacteria. *Microb. Ecol.* 71 131–149. 10.1007/s00248-015-0704-6 26582318

[B46] Taubert M. Grob C. Howat A. M. Burns O. J. Pratscher J. Jehmlich N. (2017). Methylamine as a nitrogen source for microorganisms from a coastal marine environment. *Environ. Microbiol.* 19 2246–2257. 10.1111/1462-2920.13709 28244196

[B47] Thiel V. Tank M. Bryant D. A. (2018). Diversity of chlorophototrophic bacteria revealed in the omics era. *Annu. Rev. Plant Biol.* 69 21–49. 10.1146/annurev-arplant-042817-040500 29505738

[B48] Van Goethem M. W. Makhalanyane T. P. Cowan D. A. Valverde A. (2017). Cyanobacteria and alphaproteobacteria may facilitate cooperative interactions in niche communities. *Front. Microbiol.* 8:2099. 10.3389/fmicb.2017.02099 29118751 PMC5660985

[B49] Wang H. Wei Z. Mei L. Gu J. Yin S. Faust K. (2017). Combined use of network inference tools identifies ecologically meaningful bacterial associations in a paddy soil. *Soil Biol. Biochem.* 105 227–235. 10.1016/j.soilbio.2016.11.029

[B50] Westerhuis J. A. Van Velzen E. J. J. Hoefsloot H. C. J. Smilde A. K. (2010). Multivariate paired data analysis: multilevel PLSDA versus OPLSDA. *Metabolomics* 6 119–128. 10.1007/s11306-009-0185-z 20339442 PMC2834771

[B51] Xiao B. Veste M. (2017). Moss-dominated biocrusts increase soil microbial abundance and community diversity and improve soil fertility in semi-arid climates on the Loess Plateau of China. *Appl. Soil Ecol.* 117 165–177. 10.1016/j.apsoil.2017.05.005

[B52] Yurkov V. V. Csotonyi J. T. (2009). “New light on aerobic anoxygenic phototrophs,” in *The Purple Phototrophic Bacteria*, eds Hunter C. N. Daldal F. Thurnauer M. C. Beatty J. T. (Dordrecht: Springer Academic Publishers), 31–55. 10.1007/978-1-4020-8815-5_3

[B53] Yurkov V. Hughes E. (2017). “Aerobic anoxygenic phototrophs: four decades of mystery,” in *Modern Topics in the Phototrophic Prokaryotes*, ed. Hallenbeck P. C. (Cham: Springer), 193–214.

[B54] Yutin N. Suzuki M. T. Béjà O. (2005). Novel primers reveal wider diversity among marine aerobic anoxygenic phototrophs. *Appl. Environ. Microbiol.* 71 8958–8962. 10.1128/AEM.71.12.8958-8962.2005 16332899 PMC1317425

[B55] Zeng Y. Feng F. Medová H. Dean J. Koblizek M. (2014). Functional type-2 photosynthetic reaction centers found in the rare bacterial phylum gemmatimonadetes. *Proc. Natl. Acad. Sci. U.S.A.* 111 7795–7800. 10.1073/pnas.1400295111 24821787 PMC4040607

[B56] Zeng Y. H. Chen X. H. Jiao N. Z. (2007). Genetic diversity assessment of anoxygenic photosynthetic bacteria by distance based grouping analysis of *pufM* sequences. *Lett. Appl. Microbiol.* 45 639–645. 10.1111/j.1472-765X.2007.02247.x 17922815

[B57] Zhang X. X. Liu Y. P. Yuan B. Zhao J. R. Wang R. G. Feng F. Y. (2015). *Sphingomonas* sp. MIM37 possessing aerobic anoxygenic photosynthetic gene cluster and xanthorhodopsin-like gene: its genome draft and growth stimulation by illumination. *Microbiol. Chin.* 42 1520–1528. 10.13344/j.microbiol.china.140863

[B58] Zheng Z. Dong W. Li Z. Zhao W. Hu S. Yan X. (2015). Observational study of surface spectral radiation and corresponding albedo over Gobi, desert, and bare loess surfaces in northwestern China. *J. Geophys. Res. Atmos.* 120 883–896. 10.1002/2014JD022516

