# Supplementary material for: Aerobic Anoxygenic Phototrophic Bacteria Promote the Development of Biological Soil Crusts
Source: Front Microbiol. 2018 Nov 13;9:2715. doi: 10.3389/fmicb.2018.02715 (PMC6243035; doi:10.3389/fmicb.2018.02715)
Supplement: Supplementary file 1 [file Table_1.DOCX]

**
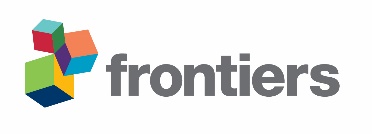
**

Supplementary Material

**Aerobic Anoxygenic Phototrophic Bacteria Promote the Development of Biological Soil Crusts**

Kai Tang^1^, Lijuan Jia^1^, Bo Yuan^1, 2^, Shanshan Yang^1^, Heng Li^1^, Jianyu Meng^1^, Yonghui Zeng^3,4^*, Fuying Feng^1^*

*Correspondence: Yonghui Zeng, [yonghui.sci@gmail.com](mailto:yonghui.sci@gmail.com) or Fuying Feng, [foyefeng@hotmail.com](mailto:foyefeng@hotmail.com)

1. **Supplementary Figures**

Red+2NIR

Red+NIR

Red+1/2NIR

30 days 75 days

**Figure S1** Visible changes on the surface soils incubated under three different levels of NIR. The digital photographs showed the apparent changes of biological soil crusts (BSCs) at day 30 and 75. Red: red light; NIR: near-infrared light. Red, range from 560 to 700 nm, peak light wavelengths at 630 and 660 nm; NIR, range from 700 to 1000 nm, peak light wavelengths at 740, 840 and 940 nm; Red+NIR, range from 560 to 1000 nm, peak light wavelengths at 630, 660, 740, 840 and 940 nm. 1/2NIR, NIR and 2NIR represents the luminous intensity at approximately 35, 70 and 140 lux, respectively.

**
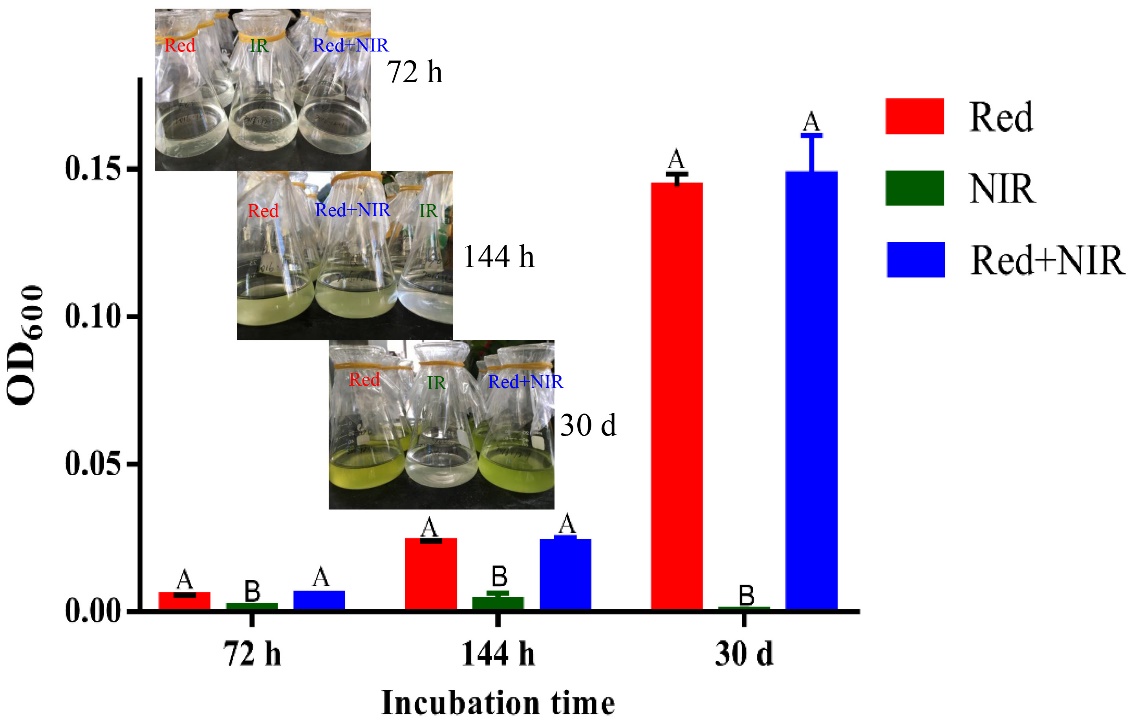
**

**Figure S2.** Effect of different wavelength lights (Red, NIR and Red+NIR) on the growth of *Microcolus vaginatus* strain MDM25 (Cyanobacteria). Red, range from 560 to 700 nm, peak light wavelengths at 630 and 660 nm; NIR, range from 700 to 1000 nm, peak light wavelengths at 740, 840 and 940 nm; Red+NIR, range from 560 to 1000 nm, peak light wavelengths at 630, 660, 740, 840 and 940 nm.

1. **Supplementary Tables**

**Table S1** PCR primers and thermal cycling conditions used for quantification of different genes.

| **Gene designation** | **Targeted group** | **Primer sequences (5'-3')** | **Temperature profile** | **Standard curve** |
| --- | --- | --- | --- | --- |
| **16S rDNA^1^** 1369F 1541R | Bacteria | CGGTGAATACGTTCYCGG AAGGAGGTGATCCRGCCGCA | 95 °C, 1 min, 1 cycle  95 °C for 15 s, 58 °C for 20 s, 72 °C for 30 s, 40 cycles 95 °C for 15 s, 60 to 95 °C, 1 cycle | y=-0.3196x+13.578 R^2^=0.9996 |
|  |  |  |  |  |
|  |  |  |  |  |
| ***puf*M^2^**  *puf*M 557F *puf*M_WAW | AAPB | TACGGSAACCTGTWCTAC CCATSGTCCAGCGCCAGAA | 95 °C, 30s,1 cycle  95 °C for 10 s, 52 °C for 15 s, 72 °C for 30 s, 40 cycles 95 °C for 15 s, 60 to 95 °C, 1 cycle | y=-0.3137x+12.504 R^2^=0.9982 |
|  |  |  |  |  |
|  |  |  |  |  |
| ***nif*H^3^**  polF polR | Azotobacter | TGCGAYCCSAARGCBGACTC ATSGCCATCATYTCRCCGGA | 95 °C, 1 min, 1 cycle  95 °C for 15 s, 58 °C for 20 s, 72 °C for 30 s, 40 cycles 95 °C for 15 s, 60 to 95 °C, 1 cycle | y=-0.2926x+12.169 R^2^=0.9973 |
|  |  |  |  |  |
|  |  |  |  |  |
| **18S rRNA^4^** 0817F 1196r | Fungus | TTAGCATGGAATAATRRAATAGGA TCTGGACCTGGTGAGTTTCC | 95 °C, 5 min, 1 cycle  95 °C for 30 s, 58 °C for 30 s, 72 °C for 45 s, 40 cycles 95 °C for 30 s, 60 to 95 °C, 1 cycle | y=-0.3003x+11.681 R^2^=0.9999 |
|  |  |  |  |  |
|  |  |  |  |  |
| **16S rDNA^5^** CYA359 CYA781RA+B | Cyanobacteria | GGGGAATYTTCCGCAATGGG  A: GACTACTGGGGTATCTAATCCCATT  B: GACTACAGGGGTATCTAATCCCTTT | 95 °C, 3 min, 1 cycle  95 °C for 30 s, 58 °C for 30 s, 72 °C for 30 s, 40 cycles 95 °C for 30 s, 60 to 95 °C, 1 cycle | y=-0.3066x+11.942 R^2^=0.9995 |
|  |  |  |  |  |
|  |  |  |  |  |
| **18S rDNA^6^**  V8F 1510R | Microalgae | ATAACAGGTCTGTGATGCCCT CCTTCYGCAGGTTCACCTAC | 95 °C, 3 min, 1 cycle  95 °C for 30 s, 58 °C for 30 s, 72 °C for 30 s, 40 cycles 95 °C for 30 s, 60 to 95 °C, 1 cycle | y=-0.3021x+11.471  R^2^=0.997 |
|  |  |  |  |  |
|  |  |  |  |  |

1. Irianni-Renno, M., Akhbari, D., Olson, M.R., Byrne, A.P., Lefèvre, E., Zimbron, J., et al. (2016). Comparison of bacterial and archaeal communities in depth-resolved zones in an LNAPL body. *Appl Microbiol Biotechnol*. 100, 3347-3360. doi: 10.1007/s00253-015-7106-z

2. Achenbach, L.A., Carey, J. and Madigan, M.T. (2001). Photosynthetic and phylogenetic primers for detection of anoxygenic phototrophs in natural environments. *Appl Environ Microbiol*. 67, 2922-2926. doi: 10.1128/AEM.67.7.2922-2926.2001

3. Brankatschk, R., Bodenhausen, N., Zeyer, J. and Bürgmann, H. (2012). Simple absolute quantification method correcting for quantitative PCR efficiency variations for microbial community samples. *Appl Environ Microbiol*. 78, 4481-4489. doi: 10.1128/AEM.07878-11

4. Wu, Z., Wang, X.R. and Blomquist, G. (2002). Evaluation of PCR primers and PCR conditions for specific detection of common airborne fungi. *J. Environ. Monit*. 4, 377-382. doi: 10.1039/B200490A

5. Steven, B., Kuske, C.R., Reed, S.C. and Belnap, J. (2015). Climate change and physical disturbance manipulations result in distinct biological soil crust communities. *Appl Environ Microbiol*. 81, 7448-7459. doi: 10.1128/AEM.01443-15

6. Bradley, I.M., Pinto, A.J. and Guest, J.S. (2016). Design and evaluation of Illumina MiSeq-compatible, 18S rRNA gene-specific primers for improved characterization of mixed phototrophic communities. *Appl Environ Microbiol*. 82, 5878-5891. doi: 10.1128/AEM.01630-16

**Table S2** Physiochemical properties of biological soil crusts, surface soil and subsoils under Red, NIR and Red+NIR illumination respectively (the small letter of “s” associated with Red, NIR or Red+NIR indicates the corresponding subsoils). Data are means ± standard error. Letters (a, b or c) in superscript indicate significant difference (*Turkey’s test; P < 0.05*). AP, available phosphorous; AN, available nitrogen; OM, organic matter; Chl *a*: Chlorophyll *a*. *N.D.*, not detected.

| Time (day) | Samples | pH | AP (mg⋅kg^-1^) | AN (mg⋅kg^-1^) | OM (g⋅kg^-1^) | Chl *a* (μg⋅kg^-1^) |
| --- | --- | --- | --- | --- | --- | --- |
| 15 | Red | 9.04 ± 0.10^c^ | 2.42 ± 0.04^ab^ | 11.42 ± 0.04^b^ | 0.66 ± 0.00^b^ | 4.50 ± 0.00^b^ |
|  | Reds | 9.28 ± 0.09^b^ | 2.18 ± 0.00^c^ | 5.13 ± 0.03^e^ | 0.58 ± 0.01^c^ | *N.D.* |
|  | NIR | 9.22 ± 0.03^b^ | 2.34 ± 0.02^b^ | 12.66 ± 0.03^a^ | 0.04 ± 0.00^e^ | *N.D.* |
|  | NIRs | 9.31 ± 0.05^b^ | 2.51 ± 0.07^a^ | 6.53 ± 0.02^c^ | 0.20 ± 0.00^d^ | *N.D.* |
|  | Red+NIR | 9.24 ± 0.03^b^ | 2.22 ± 0.06^c^ | 5.29 ± 0.02^d^ | 0.81 ± 0.00^a^ | 5.20 ± 0.00^a^ |
|  | Red+NIRs | 9.43 ± 0.03^a^ | 2.04 ± 0.06^d^ | 2.16 ± 0.02^f^ | 0.66 ± 0.01^b^ | *N.D.* |
| 45 | Red | 8.90 ± 0.05^c^ | 4.40 ± 0.05^ab^ | 20.44 ± 1.75^b^ | 1.66 ± 0.03^b^ | 6.40 ± 0.00^b^ |
|  | Reds | 9.26 ± 0.04^a^ | 2.36 ± 0.07^c^ | 8.51 ± 0.58^d^ | 0.94 ± 002^c^ | *N.D.* |
|  | NIR | 9.13 ± 0.11^b^ | 4.71 ± 0.52^a^ | 26.86 ± 1.01^a^ | 0.40 ± 0.01^d^ | *N.D.* |
|  | NIRs | 9.13 ± 0.06^b^ | 2.30 ± 0.09^c^ | 2.93 ± 0.14^f^ | 0.20 ± 0.00^e^ | *N.D.* |
|  | Red+NIR | 9.30 ± 0.05^a^ | 4.01 ± 0.02^b^ | 11.69 ± 1.75^c^ | 2.81 ± 0.04^a^ | 7.20 ± 0.00^a^ |
|  | Red+NIRs | 9.34 ± 0.08^a^ | 2.24 ± 0.07^c^ | 5.26 ± 0.53^e^ | 1.66 ± 0.03^b^ | *N.D.* |
| 75 | Red | 8.67 ± 0.01^c^ | 20.85 ± 0.28^c^ | 36.75 ± 1.75^a^ | 1.72 ± 0.04^c^ | 10.03 ± 0.01^b^ |
|  | Reds | 8.72 ± 0.04^ab^ | 20.87 ± 0.20^c^ | 20.42 ± 1.01^b^ | 1.23 ± 0.06^d^ | *N.D.* |
|  | NIR | 8.66 ± 0.03^c^ | 22.38 ± 0.98^bc^ | 36.75 ± 3.5^a^ | 0.68 ± 0.01^e^ | *N.D.* |
|  | NIRs | 8.70 ± 0.03^bc^ | 22.48 ± 1.01^b^ | 19.25 ± 1.75^b^ | 0.41 ± 0.01^f^ | *N.D.* |
|  | Red+NIR | 8.59 ± 0.01^d^ | 23.48 ± 0.94^b^ | 10.5 ± 1.75^c^ | 3.95 ± 0.04^a^ | 18.04 ± 0.00^a^ |
|  | Red+NIRs | 8.76 ± 0.01^a^ | 28.40 ± 1.03^a^ | 7.58 ± 1.01^c^ | 2.32 ± 0.05^b^ | *N.D.* |
| 120 | Red | 7.67 ± 0.03^a^ | 19.05 ± 0.06^b^ | 43.63 ± 1.01^c^ | 2.98 ± 0.09^b^ | 15.10 ± 0.20^b^ |
|  | Reds | 7.48 ± 0.03^d^ | 17.99 ± 0.21^c^ | 61.72 ± 2.02^a^ | 2.20 ± 0.09^d^ | *N.D.* |
|  | NIR | 7.60 ± 0.01^b^ | 17.85 ± 0.23^c^ | 23.80 ± 1.75^d^ | 1.37 ± 0.17^e^ | *N.D.* |
|  | NIRs | 7.55 ± 0.02^c^ | 17.97 ± 0.19^c^ | 47.13 ± 1.01^b^ | 0.94 ± 0.01^f^ | *N.D.* |
|  | Red+NIR | 7.47 ± 0.02^d^ | 21.38 ± 0.25^a^ | 40.72 ± 1.01^c^ | 6.07 ± 0.09^a^ | 17.90 ± 0.24^a^ |
|  | Red+NIRs | 7.54 ± 0.05^c^ | 17.62 ± 0.06^c^ | 49.47 ± 2.67^b^ | 2.69 ± 0.10^c^ | *N.D.* |

**Table S3** Copy numbers of microorganisms in biological soil crusts and surface soils per gram sample under Red, NIR and Red+NIR illumination respectively. AAnPB, aerobic anoxygenic phototrophic bacteria. Data are means ± standard error. Data marked in different letter (a, b or c in superscript) are significantly different (Tukey’s test; *P<0.05*). *N.D*., not detected.

| Time (days) | Samples | Bacteria | AAnPB | Diazotrophs | Fungi | Cyanobacteria | Microalgae |  |
| --- | --- | --- | --- | --- | --- | --- | --- | --- |
|  |  | 16S rRNA (×10^7^) | *puf* M (×10^5^) | *nif* H(×10^4^) | 18S rRNA(×10^5^) | 16S rRNA(×10^6^) | 18S rRNA(×10^5^) |  |
| 15 | Red | 2.59±0.10^b^ | 1.71±1.47^a^ | 3.29±0.29^a^ | 2.36±0.27^b^ | 6.64±0.23^b^ | 2.83±0.07^a^ |  |
|  | NIR | 0.39±0.03^c^ | 0.12±0.01^b^ | 0.77±0.07^c^ | 0.74±0.02^c^ | *N.D.* | *N.D.* |  |
|  | Red+NIR | 3.06±0.12^a^ | 1.52±1.65^a^ | 1.68±0.21^b^ | 3.33±0.05^a^ | 10.42±0.20^a^ | 4.13±0.93^a^ |  |
| 45 | Red | 29.88±1.35^a^ | 4.82±0.10^b^ | 9.71±1.54^b^ | 9.22±0.46^a^ | 32.52±4.37^b^ | 7.47±0.18^a^ |  |
|  | NIR | 0.85±0.05^c^ | 0.12±0.00^c^ | 1.52±0.36^c^ | 3.47±0.28^c^ | *N.D.* | *N.D.* |  |
|  | Red+NIR | 25.23±1.25^b^ | 5.37±0.06^a^ | 46.52±1.74^a^ | 4.97±0.55^b^ | 70.46±5.99^a^ | 3.36±0.07^b^ |  |
| 75 | Red | 16.94±0.07^b^ | 9.89±0.58^b^ | 12.94±0.61^b^ | 43.92±3.09^b^ | 5.48±0.32^b^ | 23.11±1.12^b^ |  |
|  | NIR | 1.97±0.04^c^ | 2.31±0.05^c^ | 18.08±1.01^b^ | 2.06±0.10^c^ | *N.D.* | *N.D.* |  |
|  | Red+NIR | 83.26±1.96^a^ | 41.54±2.51^a^ | 95.27±1.58^a^ | 533.23±4.14^a^ | 30.26±5.57^a^ | 575.91±18.67^a^ |  |
| 120 | Red | 7.93±0.40^b^ | 5.48±0.08^b^ | 23.20±2.19^b^ | 56.20±6.14^b^ | 5.78±0.63^b^ | 26.39±2.68^b^ |  |
|  | NIR | 0.40±0.04^c^ | 0.64±0.03^c^ | 8.00±0.36^c^ | 1.37±0.12^c^ | *N.D.* | *N.D.* |  |
|  | Red+NIR | 60.08±1.35^a^ | 15.09±0.17^a^ | 78.16±7.23^a^ | 479.88±4.16^a^ | 57.83±5.05^a^ | 408.40±32.13^a^ |  |

**Table S4** Diversity indices of aerobic anoxygenic phototrophic bacteria (AAnPB) based on *puf*M sequences.

| Sampling time (day) | Samples | OTU | Coverage (%) | Diversity indices | | |
| --- | --- | --- | --- | --- | --- | --- |
|  |  |  |  | Shannon | Ace | Chao1 |
| 15 | Red | 123 | 99.88 | 2.24 | 166.31 | 173.75 |
|  | NIR | 65 | 99.94 | 2.04 | 93.34 | 96.14 |
|  |  |  |  |  |  |  |
|  | Red+NIR | 110 | 99.92 | 3.52 | 139.27 | 145.14 |
|  |  |  |  |  |  |  |
| 45 | Red | 116 | 99.96 | 2.60 | 141.55 | 141.21 |
|  |  |  |  |  |  |  |
|  | NIR | 76 | 99.99 | 2.40 | 109.27 | 111.00 |
|  |  |  |  |  |  |  |
|  | Red+NIR | 95 | 99.98 | 2.01 | 138.69 | 136.67 |
|  |  |  |  |  |  |  |
| 75 | Red | 123 | 99.99 | 2.68 | 145.24 | 144.15 |
|  |  |  |  |  |  |  |
|  | NIR | 34 | 99.97 | 1.08 | 104.70 | 61.00 |
|  |  |  |  |  |  |  |
|  | Red+NIR | 121 | 99.97 | 2.58 | 148.85 | 148.00 |
|  |  |  |  |  |  |  |
| 120 | Red | 150 | 99.96 | 2.48 | 179.16 | 181.00 |
|  |  |  |  |  |  |  |
|  | NIR | 48 | 99.96 | 1.51 | 108.82 | 91.25 |
|  |  |  |  |  |  |  |
|  | Red+NIR | 136 | 99.96 | 2.68 | 167.40 | 167.23 |
|  |  |  |  |  |  |  |

**Table S5** The significant correlation (*P*<0.05) between the members of AAnPB community and soil properties, measured as the Pearson’s correlation coefficient (r).

|  | pH | AP | AN | Chl *a* | | | |
| --- | --- | --- | --- | --- | --- | --- | --- |
|  | *Methyloversatilis* | *Methyloversatilis* | *Methyloversatilis* | *Bradyrhizobium* | *Methyloversatilis* | *Skermanella* | *unclassified Proteobacteria* |
| *R* -value | -0.7291 | 0.7142 | 0.6245 | 0.7585 | 0.9763 | -0.7423 | 0.7358 |
| *P* | 0.0071 | 0.0005 | 0.0299 | 0.0291 | <0.0001 | 0.0349 | 0.0374 |

|  | OM | | | | | | |
| --- | --- | --- | --- | --- | --- | --- | --- |
|  | *Bradyrhizobium* | *Mongoliimonas* | *Novosphingobium* | *Roseomonas* | *Sphingomonas* | *unclassified Gemmatimonadetes* | *unclassified Proteobacteria* |
| *R -value* | 0.6916 | 0.6121 | 0.7259 | 0.6759 | 0.7006 | 0.7431 | 0.8746 |
| *P* | 0.0127 | 0.0344 | 0.0075 | 0.0158 | 0.0112 | 0.0056 | 0.0002 |

**Table S6** Network details of 49 OTUs identified as having significant pairwise interactions. Shades of blue and orange showed the hubs in the Alpha- and Beta-proteobacteria level, respectively. a, b, c, d means the cultivation days of 15, 45, 75, 120 under the Red+NIR.

| **OTU** | **degree** | **Closeness Centrality** | **Betweenness Centrality** | **abundance** | **aRed+NIR** | **bRed+NIR** | **cRed+NIR** | **dRed+NIR** | **Taxonomy** | [**E value**](https://blast.ncbi.nlm.nih.gov/Blast.cgi?CMD=Get&ALIGNDB_BATCH_ID=13973117&ALIGNDB_CGI_HOST=blast.st-va.ncbi.nlm.nih.gov&ALIGNDB_CGI_PATH=/ALIGNDB/alndb_asn.cgi&ALIGNDB_MASTER_ALIAS=SD_ALIGNDB_MASTER&ALIGNDB_MAX_ROWS=100&ALIGNDB_MULTIQUERY=TRUE&ALIGNDB_ORDER_CLAUSE=seq_evalue%20asc,aln_id%20asc&ALIGNDB_WHERE_CLAUSE=seq_evalue%20is%20not%20null%20and%20seq1_name=%27lcl\|Query_200765%27&ALIGNMENTS=100&ALIGNMENT_VIEW=Pairwise&CURR_QUERY_ID=lcl\|Query_200765&DATABASE_SORT=0&DESCRIPTIONS=100&DYNAMIC_FORMAT=on&FIRST_QUERY_NUM=0&FORMAT_OBJECT=Alignment&FORMAT_PAGE_TARGET=&FORMAT_TYPE=HTML&GET_SEQUENCE=yes&I_THRESH=&LINE_LENGTH=60&MASK_CHAR=2&MASK_COLOR=1&NEW_VIEW=yes&NUM_OVERVIEW=100&NUM_QUERIES=49&PAGE=Translations&QUERY_INDEX=0&QUERY_NUMBER=0&RESULTS_PAGE_TARGET=&RID=WD21E4N601N&SHOW_LINKOUT=yes&SHOW_OVERVIEW=yes&STEP_NUMBER=&USE_ALIGNDB=true&WORD_SIZE=6&OLD_VIEW=false&DISPLAY_SORT=0&HSP_SORT=0) | [**Ident**](https://blast.ncbi.nlm.nih.gov/Blast.cgi?CMD=Get&ALIGNDB_BATCH_ID=13973117&ALIGNDB_CGI_HOST=blast.st-va.ncbi.nlm.nih.gov&ALIGNDB_CGI_PATH=/ALIGNDB/alndb_asn.cgi&ALIGNDB_MASTER_ALIAS=SD_ALIGNDB_MASTER&ALIGNDB_MAX_ROWS=100&ALIGNDB_MULTIQUERY=TRUE&ALIGNDB_ORDER_CLAUSE=seq_evalue%20asc,aln_id%20asc&ALIGNDB_WHERE_CLAUSE=seq_evalue%20is%20not%20null%20and%20seq1_name=%27lcl\|Query_200765%27&ALIGNMENTS=100&ALIGNMENT_VIEW=Pairwise&CURR_QUERY_ID=lcl\|Query_200765&DATABASE_SORT=0&DESCRIPTIONS=100&DYNAMIC_FORMAT=on&FIRST_QUERY_NUM=0&FORMAT_OBJECT=Alignment&FORMAT_PAGE_TARGET=&FORMAT_TYPE=HTML&GET_SEQUENCE=yes&I_THRESH=&LINE_LENGTH=60&MASK_CHAR=2&MASK_COLOR=1&NEW_VIEW=yes&NUM_OVERVIEW=100&NUM_QUERIES=49&PAGE=Translations&QUERY_INDEX=0&QUERY_NUMBER=0&RESULTS_PAGE_TARGET=&RID=WD21E4N601N&SHOW_LINKOUT=yes&SHOW_OVERVIEW=yes&STEP_NUMBER=&USE_ALIGNDB=true&WORD_SIZE=6&DISPLAY_SORT=3&HSP_SORT=3)**ity** | **Accession number** |
| --- | --- | --- | --- | --- | --- | --- | --- | --- | --- | --- | --- | --- |
| OTU106 | 6 | 0.46 | 0.02 | 0.04 | 224 | 82 | 217 | 239 | g_uncultured bacterium | 8E-53 | 94% | AGR87560 |
| OTU135 | 7 | 0.43 | 0.02 | 0 | 2 | 3 | 9 | 4 | c_Alphaproteobacteria;o_Sphingomonadales;f_Sphingomonadaceae;g_Sphingomonas | 6E-49 | 92% | WP_066482748 |
| OTU140 | 12 | 0.47 | 0.04 | 0.14 | 284 | 90 | 908 | 1506 | p_Proteobacteria;c_;o_;f_;g_ | 2E-52 | 97% | OQW47717 |
| OTU142 | 4 | 0.39 | 0.02 | 0.07 | 585 | 479 | 253 | 127 | c_Alphaproteobacteria;o_Sphingomonadales;f_Sphingomonadaceae;g_Sphingomonas | 6E-49 | 92% | WP_066482748 |
| OTU147 | 12 | 0.5 | 0.05 | 0.01 | 40 | 2 | 118 | 87 | c_Alphaproteobacteria;o_Rhizobiales;f_Methylobacteriaceae;g_Methylobacterium | 1E-52 | 97% | WP_055886173 |
| OTU149 | 11 | 0.48 | 0.03 | 0.02 | 22 | 216 | 28 | 42 | c_Alphaproteobacteria;o_Sphingomonadales;f_Sphingomonadaceae;g_Sphingomonas | 1E-49 | 91% | WP_096342034 |
| OTU151 | 8 | 0.43 | 0.03 | 0.57 | 462 | 660 | 3929 | 6581 | c_Betaproteobacteria;o_Nitrosomonadales;f_Sterolibacteriaceae;g_Methyloversatilis | 3E-53 | 100% | EGK69993 |
| OTU161 | 1 | 0.28 | 0 | 0 | 10 | 14 | 19 | 5 | c_Betaproteobacteria;o_Burkholderiales;f_Comamonadaceae;g_ | 9E-54 | 100% | WP_056182925 |
| OTU163 | 12 | 0.51 | 0.05 | 0.03 | 110 | 2 | 312 | 164 | c_Alphaproteobacteria;o_Sphingomonadales;f_Sphingomonadaceae;g_Novosphingobium | 2E-53 | 99% | WP_054106410 |
| OTU173 | 17 | 0.49 | 0.03 | 0.02 | 261 | 6 | 26 | 28 | c_Betaproteobacteria;o_Burkholderiales;f_Comamonadaceae;g_ | 9E-54 | 100% | WP_056182925 |
| OTU175 | 6 | 0.42 | 0 | 0.01 | 94 | 15 | 27 | 3 | c_Betaproteobacteria;o_Burkholderiales;f_Comamonadaceae;g_Rhodoferax | 3E-51 | 97% | WP_075586643 |
| OTU178 | 16 | 0.52 | 0.02 | 0.08 | 1141 | 31 | 364 | 166 | c_Alphaproteobacteria;o_Rhizobiales;f_Phyllobacteriaceae;g_Mesorhizobium | 5E-53 | 99% | WP_027033744 |
| OTU182 | 3 | 0.35 | 0 | 0 | 3 | 2 | 12 | 1 | c_Alphaproteobacteria;o_Rhizobiales;f_Phyllobacteriaceae;g_Hoeflea | 6E-49 | 89% | WP_052161573 |
| OTU186 | 11 | 0.49 | 0.03 | 0.02 | 166 | 58 | 155 | 99 | c_Betaproteobacteria;o_Burkholderiales;f_;g_ | 1E-53 | 100% | WP_056315586 |
| OTU19 | 10 | 0.41 | 0 | 0.01 | 275 | 1 | 2 | 3 | c_Alphaproteobacteria;o_Sphingomonadales;f_Sphingomonadaceae;g_Novosphingobium | 8E-54 | 100% | WP_054106410 |
| OTU190 | 14 | 0.48 | 0.03 | 0.03 | 289 | 72 | 112 | 195 | c_Alphaproteobacteria;o_Rhizobiales;f_Methylobacteriaceae;g_Methylobacterium | 1E-52 | 97% | WP_055886173 |
| OTU191 | 9 | 0.43 | 0.01 | 0 | 1 | 1 | 3 | 2 | c_Alphaproteobacteria;o_Rhizobiales;f_Methylobacteriaceae;g_Methylobacterium | 2E-55 | 100% | WP_100251218 |
| OTU195 | 12 | 0.49 | 0.05 | 0 | 9 | 2 | 26 | 51 | c_Betaproteobacteria;o_Burkholderiales;f_;g_ | 4E-51 | 95% | WP_056315586 |
| OTU197 | 12 | 0.45 | 0.04 | 0.01 | 6 | 1 | 36 | 62 | c_Betaproteobacteria;o_Burkholderiales;f_;g_ | 6E-53 | 99% | WP_056315586 |
| OTU2 | 7 | 0.39 | 0.01 | 0.15 | 1010 | 1553 | 388 | 19 | c_Alphaproteobacteria;o_Rhodobacterales;f_;g_ | 5E-54 | 100% | OYX18913 |
| OTU20 | 7 | 0.45 | 0.03 | 0.02 | 367 | 71 | 4 | 6 | c_Betaproteobacteria;o_;f_;g_ | 2E-50 | 92% | WP_054152609 |
| OTU200 | 17 | 0.51 | 0.03 | 0.09 | 1308 | 46 | 194 | 235 | c_Alphaproteobacteria;o_Sphingomonadales;f_Sphingomonadaceae;g_Sphingomonas | 6E-49 | 92% | WP_066482748 |
| OTU202 | 5 | 0.38 | 0.01 | 0.03 | 131 | 104 | 278 | 105 | c_Betaproteobacteria;o_Burkholderiales;f_Comamonadaceae;g_Limnohabitans | 7E-52 | 95% | WP_062403225 |
| OTU206 | 8 | 0.44 | 0.03 | 0.01 | 24 | 164 | 23 | 9 | c_Betaproteobacteria;o_Burkholderiales;f_Comamonadaceae;g_Hydrogenophaga | 6E-52 | 97% | WP_069046181 |
| OTU209 | 13 | 0.5 | 0.07 | 0 | 11 | 2 | 18 | 15 | c_Alphaproteobacteria;o_Rhizobiales;f_Bradyrhizobiaceae;g_Bradyrhizobium | 1E-51 | 95% | WP_061025526 |
| OTU21 | 6 | 0.37 | 0 | 0.68 | 2748 | 11120 | 81 | 15 | c_Betaproteobacteria;o_Burkholderiales;f_;g_Methylibium | 1E-52 | 97% | KNZ32832 |
| OTU210 | 8 | 0.4 | 0.02 | 0.01 | 32 | 5 | 201 | 24 | c_Betaproteobacteria;o_Burkholderiales;f_Burkholderiaceae;g_Polynucleobacter | 9E-52 | 97% | WP_046330415 |
| OTU213 | 15 | 0.49 | 0.01 | 0.03 | 405 | 5 | 124 | 44 | c_Alphaproteobacteria;o_Sphingomonadales;f_Sphingomonadaceae;g_Sphingomonas | 7E-52 | 98% | WP_084184326 |
| OTU219 | 6 | 0.45 | 0.04 | 0 | 18 | 3 | 6 | 43 | c_Alphaproteobacteria;o_Sphingomonadales;f_Sphingomonadaceae;g_Sphingomonas | 2E-51 | 93% | WP_096342034 |
| OTU221 | 3 | 0.35 | 0 | 0 | 2 | 3 | 5 | 7 | c_Betaproteobacteria;o_Burkholderiales;f_;g_Methylibium | 6E-53 | 98% | KNZ32832 |
| OTU222 | 14 | 0.51 | 0.03 | 0 | 33 | 3 | 18 | 9 | c_Betaproteobacteria;o_Nitrosomonadales;f_Sterolibacteriaceae;g_Methyloversatilis | 1E-51 | 97% | WP_069039368 |
| OTU223 | 10 | 0.48 | 0.03 | 0.01 | 53 | 7 | 62 | 177 | c_Alphaproteobacteria;o_Rhizobiales;f_Bradyrhizobiaceae;g_Bradyrhizobium | 1E-51 | 95% | WP_061025526 |
| OTU239 | 3 | 0.38 | 0 | 0.06 | 84 | 610 | 37 | 545 | c_Alphaproteobacteria;o_Sphingomonadales;f_Sphingomonadaceae;g_ | 9E-52 | 97% | WP_086115660 |
| OTU24 | 15 | 0.48 | 0.02 | 0.03 | 553 | 14 | 31 | 65 | c_Betaproteobacteria;o_Burkholderiales;f_Comamonadaceae;g_Hydrogenophaga | 1E-52 | 99% | WP_086119319 |
| OTU243 | 9 | 0.4 | 0 | 0.15 | 1612 | 14 | 82 | 1355 | c_Alphaproteobacteria;o_Sphingomonadales;f_Sphingomonadaceae;g_ | 1E-48 | 90% | WP_086115660 |
| OTU259 | 11 | 0.43 | 0.02 | 0.01 | 26 | 99 | 52 | 31 | c_Betaproteobacteria;o_Burkholderiales;f_;g_ | 1E-51 | 97% | WP_056315586 |
| OTU264 | 11 | 0.45 | 0.01 | 0.15 | 1423 | 347 | 436 | 865 | c_Alphaproteobacteria;o_Sphingomonadales;f_Sphingomonadaceae;g_ | 1E-48 | 90% | WP_086115660 |
| OTU269 | 13 | 0.52 | 0.03 | 0.04 | 292 | 54 | 262 | 243 | c_Alphaproteobacteria;o_Sphingomonadales;f_Sphingomonadaceae;g_Novosphingobium | 1E-53 | 99% | WP_054106410 |
| OTU276 | 14 | 0.48 | 0.04 | 0.03 | 475 | 1 | 68 | 10 | c_Alphaproteobacteria;o_Sphingomonadales;f_Sphingomonadaceae;g_Novosphingobium | 8E-54 | 100% | WP_054106410 |
| OTU277 | 15 | 0.49 | 0.02 | 0.01 | 102 | 2 | 23 | 5 | c_Alphaproteobacteria;o_Sphingomonadales;f_Sphingomonadaceae;g_Sphingomonas | 6E-49 | 92% | WP_066482748 |
| OTU283 | 16 | 0.52 | 0.07 | 0.01 | 116 | 11 | 14 | 43 | c_Alphaproteobacteria;o_Rhizobiales;f_Methylobacteriaceae;g_Methylobacterium | 1E-52 | 97% | WP_055886173 |
| OTU285 | 15 | 0.55 | 0.07 | 0.03 | 271 | 11 | 205 | 202 | g_uncultured bacterium | 5E-52 | 91% | AGT59300 |
| OTU298 | 4 | 0.39 | 0.04 | 0 | 15 | 7 | 5 | 19 | g_uncultured bacterium | 6E-52 | 93% | ABW80213 |
| OTU303 | 9 | 0.48 | 0.03 | 0.78 | 1428 | 1684 | 8176 | 4398 | c_Betaproteobacteria;o_Nitrosomonadales;f_Sterolibacteriaceae;g_Methyloversatilis | 3E-53 | 100% | EGK69993 |
| OTU308 | 4 | 0.37 | 0 | 0 | 3 | 4 | 1 | 3 | c_Alphaproteobacteria;o_Rhizobiales;f_Bradyrhizobiaceae;g_Bradyrhizobium | 1E-51 | 95% | WP_061025526 |
| OTU316 | 15 | 0.55 | 0.06 | 0.01 | 38 | 3 | 36 | 24 | c_Alphaproteobacteria;o_Rhizobiales;f_Bradyrhizobiaceae;g_Bradyrhizobium | 7E-50 | 91% | WP_061025526 |
| OTU321 | 15 | 0.48 | 0.02 | 0.03 | 341 | 15 | 34 | 170 | c_Alphaproteobacteria;o_Sphingomonadales;f_Sphingomonadaceae;g_ | 1E-48 | 90% | WP_086115660 |
| OTU5 | 4 | 0.39 | 0.01 | 0.19 | 415 | 2260 | 995 | 203 | c_Alphaproteobacteria;o_Rhizobiales;f_Hyphomicrobiaceae;g_Hyphomicrobium | 3E-54 | 100% | WP_020086078 |
| OTU60 | 11 | 0.51 | 0.08 | 0.02 | 227 | 64 | 18 | 54 | c_Alphaproteobacteria;o_Rhodospirillales;f_Rhodospirillaceae;g_Skermanella | 1E-53 | 100% | WP_037453850 |
